# Mutation of the highly conserved amino acids in the N-terminal region of the EV-A71 VP1 protein impairs viral RNA release during virus entry

**DOI:** 10.1128/spectrum.02169-25

**Published:** 2026-01-16

**Authors:** Wenjing Zhang, Yaru Liu, Quanjie Li, Dongrong Yi, Qian Liu, Saisai Guo, Jing Wang, Jianyuan Zhao, Ling Ma, Jiwei Ding, Rui Zhou, Yongcheng Ren, Yesem Talpbek, Xiaoyu Li, Yongxin Zhang, Shan Cen

**Affiliations:** 1 Institute of Medicinal Biotechnology, Chinese Academy of Medical Sciences & Peking Union Medical College 71040, Beijing, China; 2 Xinjiang Key Laboratory of Uygur Medical Research, Xinjiang Institute of Materia Medica 643339, Urumqi, China; Oklahoma State University College of Veterinary Medicine, Stillwater, Oklahoma, USA

**Keywords:** EV-A71, VP1, N-terminal region, highly conserved residues, uncoating, viral RNA release

## Abstract

Enterovirus A71 (EV-A71) causes hand-foot-mouth disease, which may progress to severe neurological and systemic complications in young children and infants. To deliver viral genomic RNA into the target cell for replication, the mature EV-A71 capsid particle undergoes drastic structural changes to become an expanded A particle, which is marked by the movement of the N-terminal region of viral protein VP1 from the interior to the capsid surface and the dislodgement of the pocket factor. It is not completely understood which amino acids support these key activities of VP1 during EV-A71 entry. To answer this question, we investigated the functions of amino acids that are highly conserved in VP1 across all genotypes of EV-A71 by performing reverse genetic mutagenesis and functional experiments. Among the 21 highly conserved amino acids, 20 affect the replication of EV-A71. Notably, 7 of the 21 highly conserved amino acids are located in the 71-amino-acid N-terminal region, and mutation of any of these seven amino acids is lethal to EV-A71. Further mechanistic studies showed that the most detrimental defect occurred at the step of viral capsid uncoating and viral RNA release during virus entry, which is supported by the results of structural analyses showing the disruption of complex hydrogen bonding and hydrophobic networking due to mutating these amino acids. Our data have thus unraveled the genetic determinants that govern the key function of the N-terminal region of VP1 during EV-A71 entry and viral RNA release.

**IMPORTANCE:**

Hand, foot, and mouth disease (HFMD) annually affects millions of children worldwide. EV-A71 is a major cause of HFMD in the Asia-Pacific region. Notably, EV-A71 infection can lead to severe neurological syndromes and even death in young children; however, no specific vaccines or drugs are available. Among picornavirus capsid proteins, VP1 is the most external, surface-accessible, and immunodominant protein. However, it is not completely understood which amino acids support these key activities of VP1 during EV-A71 entry. Here, we report that 7 of the 21 highly conserved amino acids within the 71-amino-acid N-terminal region of VP1, and mutation of any of these 7 amino acids is lethal to EV-A71 by disrupting capsid uncoating and RNA release during entry. Overall, our findings highlight that the highly conserved N-terminal region residues of VP1 play a crucial role in viral infectivity and may contribute to the development of broad-spectrum anti-EV-A71 vaccines and drugs.

## INTRODUCTION

Enterovirus A71 (EV-A71) is one of the main causes of hand-foot-and-mouth disease (HFMD) in the Asia-Pacific region (1). Its infection can lead to severe neurological syndromes and death in young children. However, no EV-A71 vaccines or drugs are available. EV-A71 is a member of the *Enterovirus genus* within the *Picornaviridae* family (2). It has a single-stranded positive-sense RNA genome of 7.4 kb, which is packaged within the viral capsid of about 30 nm in size (3, 4). The icosahedral capsid of EV-A71 consists of 60 copies of four viral proteins VP1–VP4 that together form a protomer. VP1, VP2, and VP3 follow a pseudo T = 3 arrangement and form the shell of the capsid (5), whereas VP4 is located on the inner portion of the capsid (6). VP1 is the most external, surface-accessible, and immunodominant protein among the picornavirus capsid proteins, and its sequences have been used for *picornavirus* classification (7–9).

VP1 forms a few key structures in the viral capsid. The first is the “canyon” at the 5-fold axis of the capsid surface (9), serving as the major binding site for the key EV-A71 receptor scavenger receptor class member 2 (SCARB2) (10). This canyon is built by the VP1 surface loops BC and GH with contribution from VP2 EF loop, which are often the major epitopes for neutralizing antibodies (11–13) and are thus pressured by the host immunity to evolve, the most diversified among all viral proteins. The second key structure that VP1 forms is a hydrophobic pocket, which is occupied by a pocket factor that is lipid in nature (11, 14–16). This pocket is located right below the floor of the canyon, which in retrospect serves as the roof of the pocket (6). Occupation of the pocket by the lipid factor stabilizes the capsid (11, 14, 15, 17). Upon receptor binding to the canyon, this pocket factor is dislodged, which triggers the uncoating of the capsid and subsequent delivery of viral RNA into the target cells for replication (11, 16, 18). Thus, this pocket has become the target of EV-A71 inhibitors that resist the force of receptor binding, thus blocking capsid uncoating (19, 20).

The N-terminal region (amino acids 1–71) of VP1 does not participate in the formation of the canyon or pocket at the 5-fold axis but is rather positioned at the 2-fold axis and points toward the inner face of the capsid (21). With receptor binding and ejection of the pocket factor, the N-terminal region of VP1 moves to the surface of the capsid together with VP4 and viral RNA (22, 23). Residues 1–53 are disordered and exposed on the capsid surface, and residues 54–71 link to the inner capsid (24). This drastic change in VP1 is accompanied by a reorganization of the protomer interfaces, leading to particle expansion and the opening of 2-fold channels in the capsid, which serves as the gate for the extrusion of the viral RNA and the externalization of the VP1 N-terminus (18, 21, 25). This multistep uncoating process is characterized by the conversion of the mature capsid of 160S into an expanded intermediate particle or A-particle of 135S. Upon release of viral genomic RNA, this A-particle becomes the empty capsid of 80S (26, 27). Understanding of these molecular details of capsid uncoating of picornaviruses, including EV-A71, has benefited from the purification and structural characterization of these three types of particles.

These many essential functions of VP1 make this viral structural protein a major virulence factor of EV-A71, and some of the genetic determinants have been reported. For example, altering the amino acid at position 145 in VP1 modulates the binding to host factors PSGL-1 and heparan sulfate, thus changing the tropism and virulence of EV-A71, with VP1-145Q associated with more severe diseases in humans (28–30). We have focused on investigating the functions of amino acids in VP1 that are highly conserved among EV-A71 strains. One example is the amino acid at position 107 that is highly conserved and was shown to control EV-A71 maturation by regulating the cleavage of precursor protein VP0 (31). Mutations of the highly conserved amino acids at positions 75, 78, and 88 led to the production of abnormal EV-A71 particles that are defective in entering and replicating in the target cells (32). To systematically investigate the functions of highly conserved amino acids in VP1, we analyzed 3,777 EV-A71 strains available in the GenBank database and identified 21 amino acid residues that are 100% conserved in these strains. Each of these 21 amino acids was then mutated in the full-length genome of EV-A71 using reverse genetics and was examined for its effects on viral production and infection. Except for the S168A mutant that replicates similarly to the wild type of EV-A71, mutating 8 of the rest 20 highly conserved amino acids decreases viral titers by over 10-fold, whereas the rest 12 of these 20 highly conserved amino acids are indispensable to produce viable EV-A71 particles. Notably, 7 of these 12 indispensable amino acids are located in the N-terminal region, including T32, A46, M63, E65, T66, L70, and N71. Further mechanistic studies show that mutating these seven amino acids affects uncoating and consequently, viral RNA release. Overall, these results highlight that the N-terminal region of VP1 is more conserved than the rest of VP1, likely because of the need to support its essential function in capsid uncoating.

## RESULTS

### Three-dimensional spatial distribution of the highly conserved amino acids in VP1

**Fig 1:**
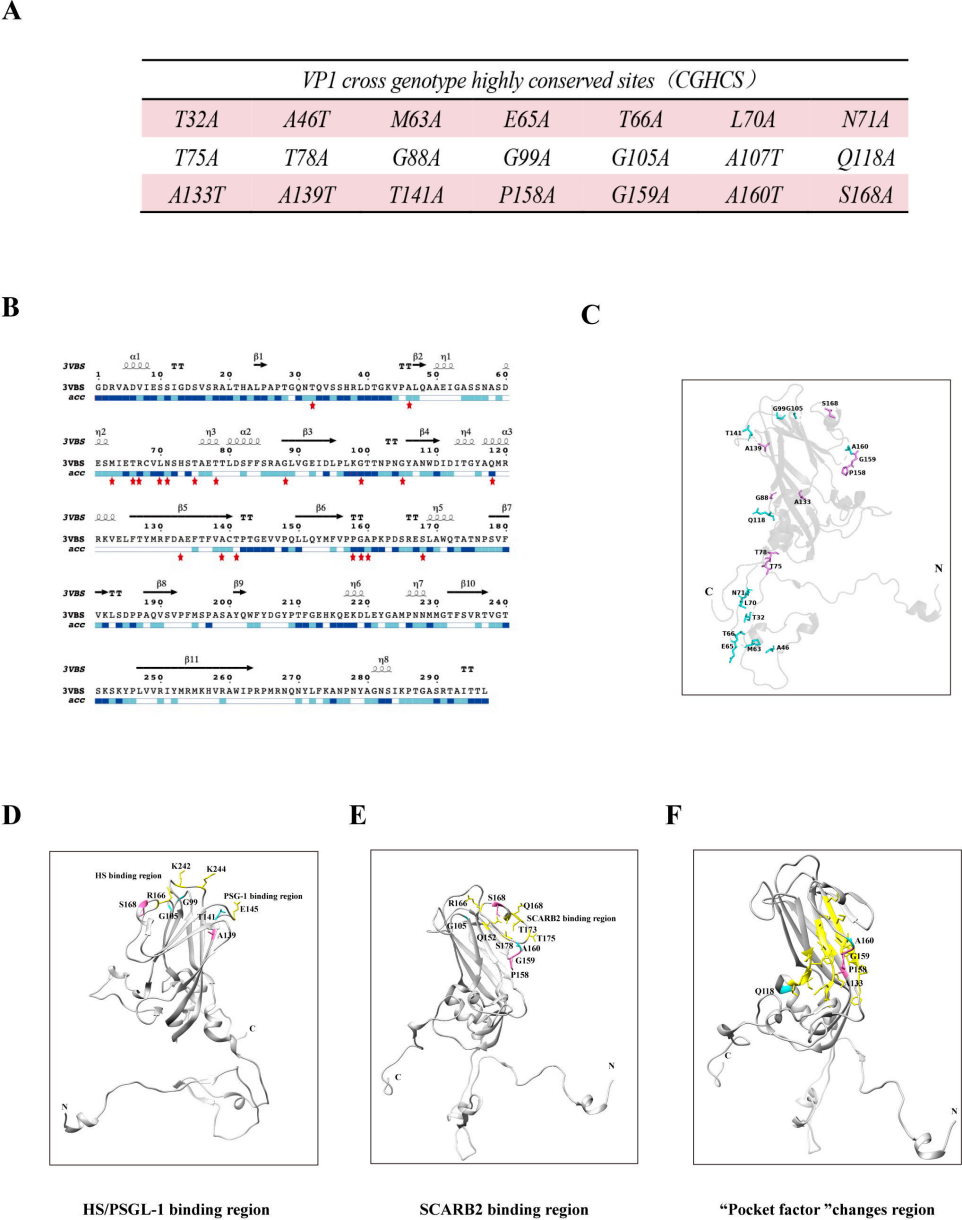
Illustration of the highly conserved amino acids in VP1. (**A**) Twenty-one highly conserved residues are identified using the ClustalW software across 3,777 EV-A71 strains. (**B**) The highly conserved amino acids in the context of the EV-A71 VP1 protein sequence. The red pentagram represents the location of mutant residues. Accessibility at the bottom indicates the accessibility of various regions. Blue shows accessible regions. Cyan shows partially accessible regions, and white shows inaccessible regions. (**C**) Depiction of the highly conserved amino acids in the atomic structure of VP1 extracted from the VP1-4 complex (PDB: 3VBS). The positions of the amino acids in the crystal structure are shown. The N and C termini are indicated. (**D–F**) Highly conserved amino acids that are located close to the binding site of the PSGL-1 or HS receptor (**D**), the SCARB2 receptor (**E**), and the pocket factor (**F**). In yellow are the VP1 regions binding to HS, PSGL-1, SCARB2, and the pocket factor. In cyan and hot pink are G99, G105, Q118, A133, A139, T141, P158, G159, A160, and S168 amino acids.

To identify potential virulence determinants in VP1, we analyzed the available VP1 protein sequences of EV-A71 strains across all three genotypes A, B, and C. The results of sequence alignment revealed 21 highly conserved amino acids in VP1, defined as “cross-genotype highly conserved sites” (CGHCS) (Fig. 1A). Notably, these 21 CGHCS are concentrated in the first two-thirds of the VP1 sequence (Fig. 1B). Accessibility analysis showed that amino acids T75, T78, A133, and T141 are buried inside, while the remaining 17 CGHCS are exposed at the protein surface. Specifically, amino acids A46, P158, and G159 are situated in the β torsion angle (TT) region, T78 and G88 are in the β3 region, Q118 is found in the α3 region, A133 and A139 are in the β5 region, and the rest of CGHCS are positioned in the N-terminal or C-terminal regions (Fig. 1B). Annotation of CGHCS in the structure of VP1 confirms that most of them are exposed at the protein surface (Fig. 1C), suggesting that they might interact with cellular and/or viral factors. Indeed, amino acids G99, G105, A139, T141, and S168 are proximal to site binding to receptors PSGL-1 or HS (Fig. 1D), G105, P158, G159, A160, and S168 are located to the binding site of receptor SCARB2 (Fig. 1E). Amino acids Q118, A133, P158, G159, and A160 are located close to the pocket structure (Fig. 1F), suggesting their roles in the binding and releasing of the pocket factor.

### The highly conserved amino acids in VP1 are essential for producing infectious EV-A71

**Fig 2:**
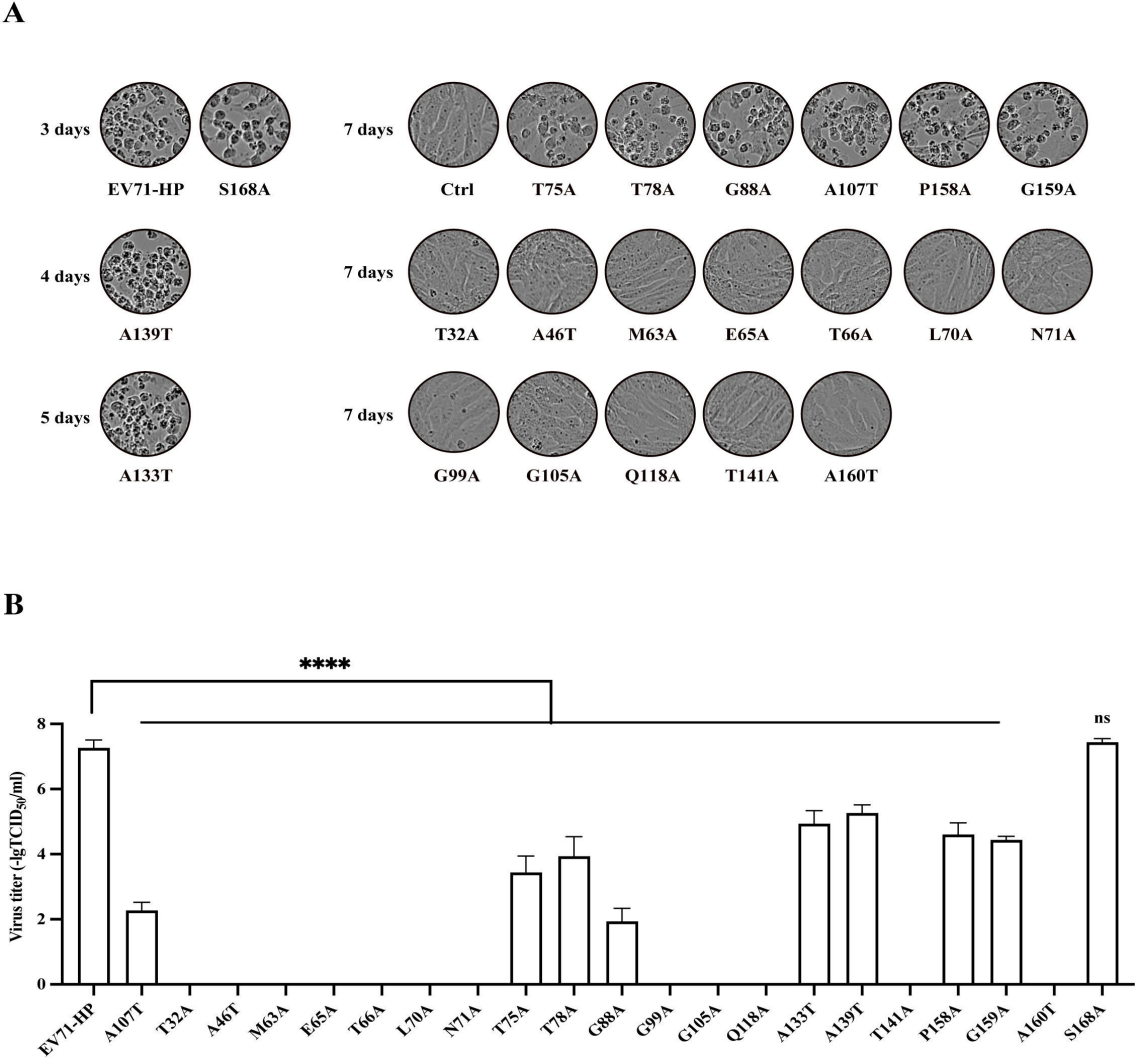
Effect of highly conserved amino acids in VP1 on the production of infectious EV-A71. (**A**) CPE of Vero cells that were transfected with 2 μg of the wild-type or mutated EV-A71 RNA over 7 days. (**B**) Titers of EV-A71 that were produced by Vero cells 48 hours after transfection with viral RNA. Viral titers were determined in the TCID_50_ assay. All experiments were performed in triplicate. At each time point, titers are the means of three samples. Error bars represent SEM. ****, *P* < 0.0001. ns, not significant.

To examine the function of these highly conserved amino acids in VP1, we mutated each of these 21 amino acids in the wild-type cDNA clone EV-A71-HP and generated a panel of viral cDNA mutants. The full-length viral RNA was *in vitro* transcribed from these DNA clones, and the same amount of wild-type and each mutated viral RNA was transfected into Vero cells to produce viral particles. The CPE was monitored daily after transfection and was observed at different times for the wild type and mutated EV-A71 (Fig. 2A). The wild-type EV-A71-HP and the S168A mutant produced evident CPE at day 3 after RNA transfection, mutants A133T and A139T displayed CPE after 4 and 5 days, respectively, mutants T75A, T78A, G88A, A107T, P158A, and G159A produced CPE until day 7, and no CPE was observed for the remaining 12 mutants (Fig. 2A). We sequenced the VP1 gene of the nine mutants when they developed CPE and confirmed the presence of the originally engineered mutations and did not detect secondary mutations. We further measured levels of infectious EV-A71 at day 2 post-transfection by performing the TCID_50_ assay. Mutant S168A produced similar titers of viruses as the wild type of EV-A71-HP, titers of mutants A133T and A139T were 100-fold lower, titers of mutants T75A, T78A, P158A, and P159A dropped by 10^3^-fold to 10^4^-fold, mutants G88A and A107T produced 10^7^-fold less viruses (Fig. 2B). No titers were detected for the 12 mutations that also did not produce CPE. It is noted that 7 of the 12 lethal mutations are located in the 71-amino-acid N-terminal region of VP1. These results demonstrate that most of these highly conserved residues of VP1 are essential for EV-A71 replication and that mutating 12 of these 21 CGHCS is lethal to the production of infectious EV-A71.

### The highly conserved amino acids in the N-terminal region of VP1 are essential for EV-A71 to replicate

**Fig 3:**
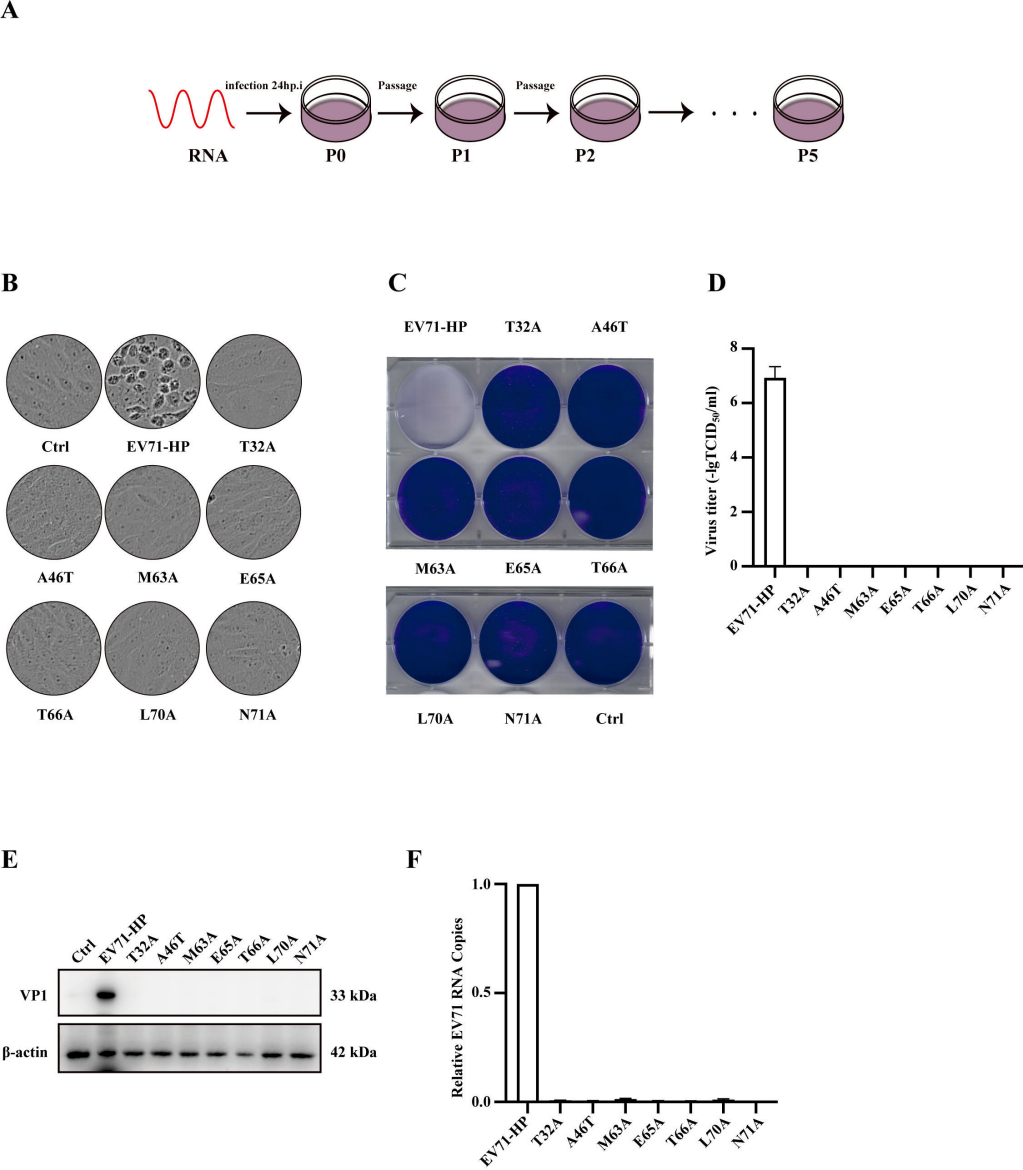
Mutants of the highly conserved amino acids in the N-terminal region of VP1 do not revert after five passages in Vero cells. (**A**) The schematic of EV-A71 passage in Vero cells. (**B**) CPE of Vero cells exposed to the wild type or mutated EV-A71 at passage 5 (P5). (**C**) Viability of infected Vero cells was visualized by staining with 0.02% crystal violet. (**D**) Titers of wild-type and mutated EV-A71 at P5. (**E**) Levels of EV-A71-VP1 protein in the lysates of Vero cells at P5. (**F**) Levels of EV-A71 RNA in the infected Vero cells at P5. All experiments were performed in triplicate. Error bars represent SEM.

Mutating any of the seven highly conserved amino acids in the 71-amino-acid N-terminal region abrogates the production of infection EV-A71, highlighting the importance of these seven amino acids in the pivotal function of this N-terminal region. To gain insights into the essential role of these seven amino acids, we passaged the mutated viruses, hoping that the virus would be able to mutate a second site in VP1 or other viral proteins to compensate for the original mutation and regain the ability to replicate. These compensatory mutations often inform the function of the original mutation. We thus passaged these seven viral mutants in Vero cells for up to five rounds, monitored CPE daily (Fig. 3A). All seven mutants failed to generate CPE by P5 (Fig. 3B), did not cause cell death (Fig. 3C), and did not produce infectious viruses (Fig. 3D). By P5, no viral protein (Fig. 3E) nor viral RNA (Fig. 3F) was detected for these seven mutants. These results suggest that mutating any of these seven highly conserved amino acids in the N-terminal region of VP1 is lethal to the virus; no viral revertant can be selected.

### The highly conserved amino acids in the N-terminal region of VP1 minimally affect viral gene expression and virus production

**Fig 4:**
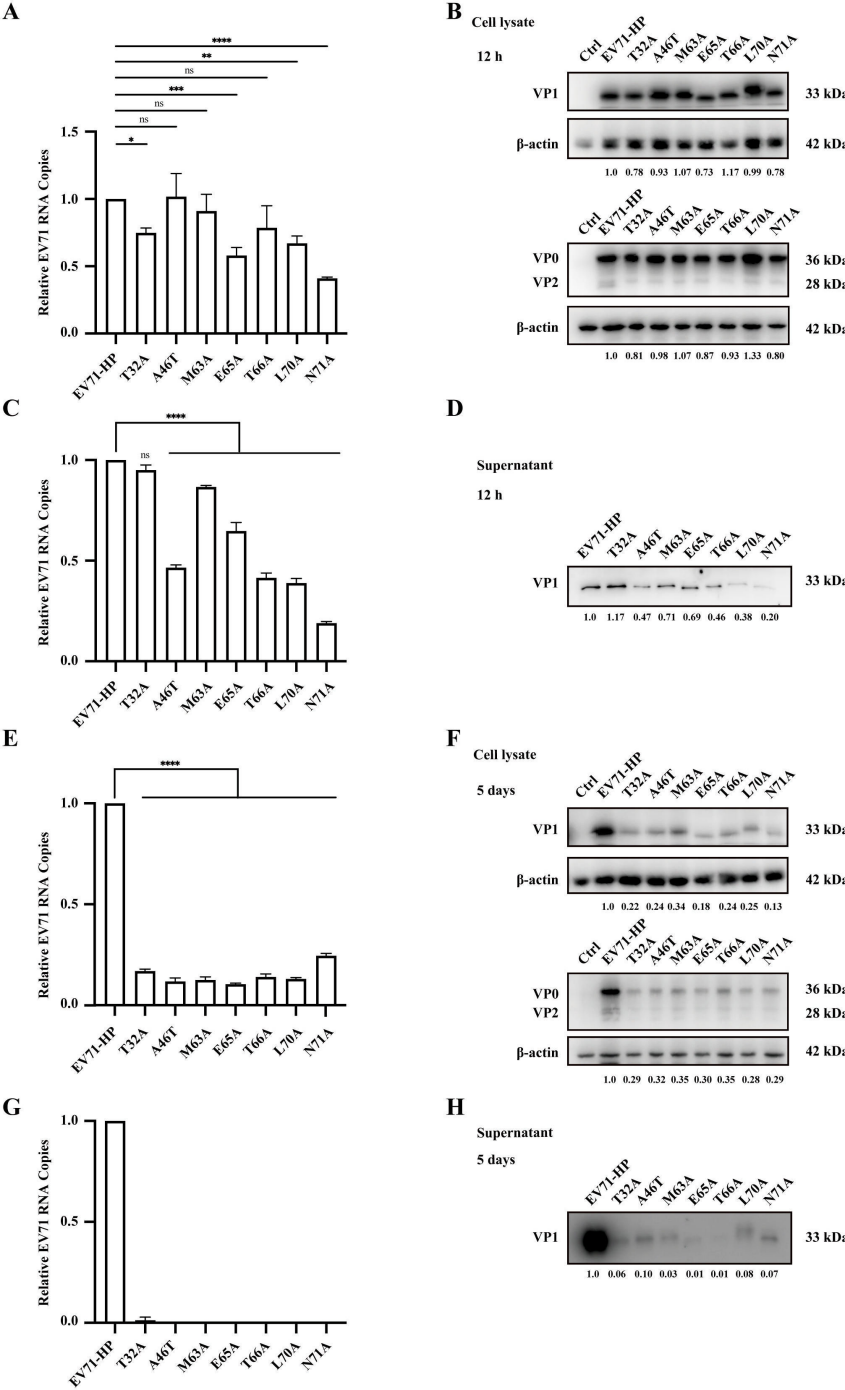
Effect of mutating the highly conserved amino acids in the N-terminal region of VP1 on viral RNA transcription, viral protein synthesis, and virus production. Vero cells were transfected with EV-A71 RNA (2 μg) for 12 h or 5 days before levels of viral RNA in cells and the supernatants were determined by RT-qPCR, and the amounts of viral proteins in cells and supernatants were examined with western blotting. (**A and B**) Levels of viral RNA and proteins in cells 12 h after transfection. (**C and D**) Levels of viral RNA and proteins in the supernatants 12 h after transfection. (**E and F**) Levels of viral RNA and proteins in cells 5 days after transfection. (**G and H**) Levels of viral RNA and proteins in the supernatants 5 days after transfection. Intensity of protein bands in the western blots was determined using ImageJ software, and the relative levels of the wild-type virus were arbitrarily set as 1. Viral particles in supernatants were collected by ultracentrifugation through a 15% sucrose cushion before being examined in Western blotting. Levels of viral RNA were determined by RT-qPCR. Error bars represent SEM. *, *P* < 0.05, **, *P* < 0.01, ***, *P* < 0.001, ****, *P* < 0.0001. ns, not significant.

To determine which stage of EV-A71 replication is abrogated by mutations of the highly conserved amino acids in the N-terminal region of VP1, we first measured viral gene transcription and translation 12 h after transfecting equal amounts of *in vitro* transcribed full-length viral RNA into Vero cells. At this time point, there is minimal secondary infection of the newly produced viral progeny. The levels of viral RNA were determined by RT-qPCR to assess viral RNA transcription; only the T71A mutant showed a more than 2-fold decrease (Fig. 4A). Western blotting was performed to measure the levels of viral proteins VP1, VP0, and VP2; none of the mutations caused a more than 2-fold decrease (Fig. 4B). We harvested EV-A71 particles in the culture supernatants and determined their amounts by Western blotting and RT-qPCR. The N71A mutation caused a 5-fold decrease; mutations A46T, T66A, and L70A caused an over 2-fold reduction in virus production (Fig. 4C and D). Therefore, mutating the highly conserved amino acids in the N-terminal region of VP1 has minimal effect on viral RNA load and viral protein synthesis, except for the N71A mutation that diminishes viral RNA by about 2-fold. A moderate reduction in virus production is caused by mutations A46T, T66A, L70A, and N71A, corroborating a role of the N-terminal region of VP1 in virus assembly. We followed up with viral gene expression and virus production until day 5 after transfection. Background levels of viral RNA and viral proteins VP1, VP0, and VP2 were detected in the transfected cells for all seven mutants (Fig. 4E and F). Residual amounts of the mutated virus particles were detected in the culture supernatants (Fig. 4G and H). These results indicate that close to wild-type levels of virus particles can be produced in the presence of the mutations of the highly conserved amino acids in the N-terminal region of VP1, but these mutations are fatal in re-infection and further spread in cultured cells. We also observed that VP1 mutants E65A, T66A, and N71A migrated faster than the wild-type VP1 and that mutant L70A migrated slower than the wild-type VP1. This suggests that these mutations may alter the conformation and folding of VP1.

### Mutating the highly conserved amino acids in the N-terminal region of VP1 blocks EV-A71 RNA release

**Fig 5:**
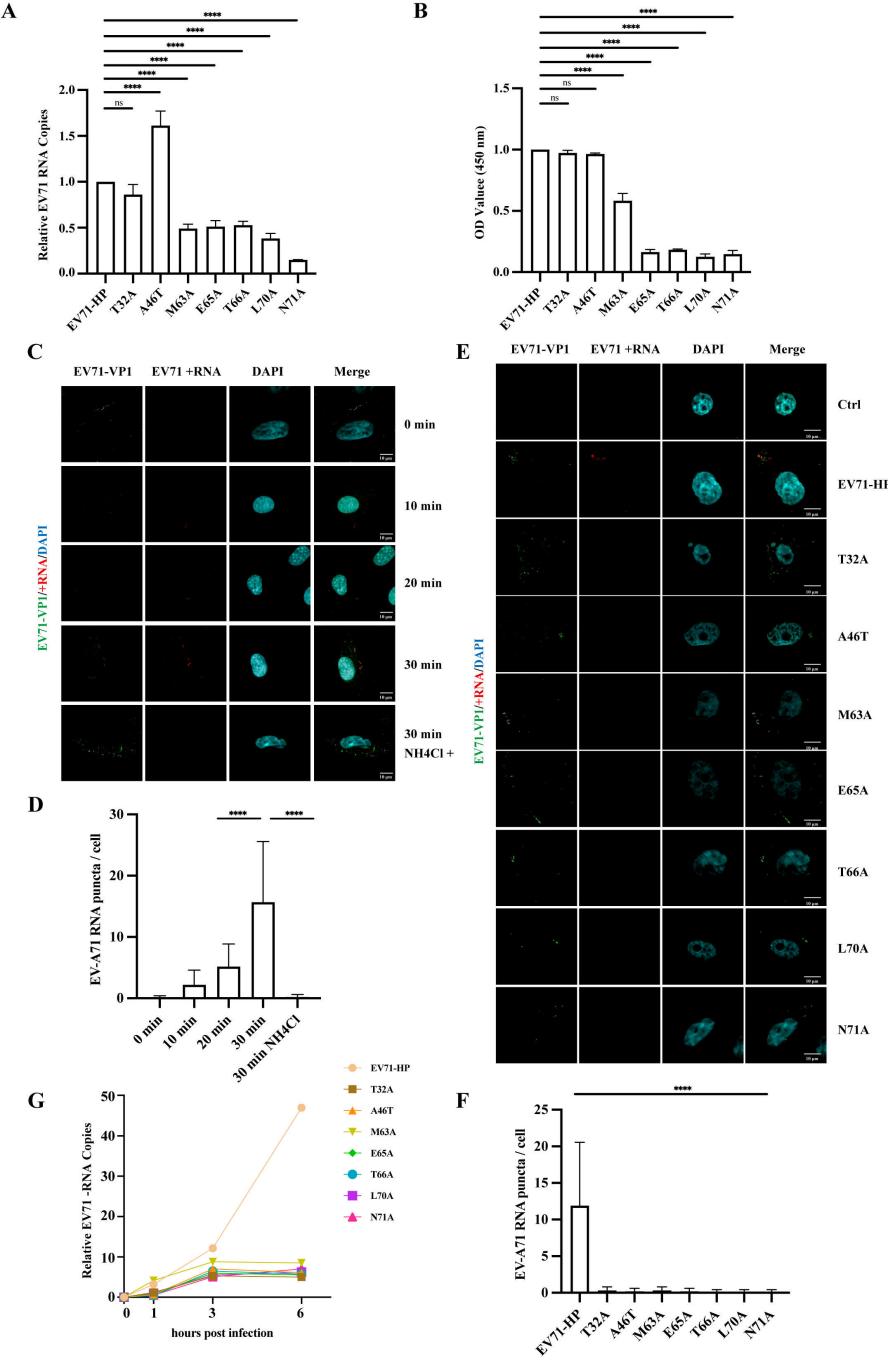
Effect of VP1 N-terminal mutations on virus attachment and viral RNA release. (**A**) Vero cells were incubated with the wild type and mutated viruses of the same amount of genome RNA at 4°C for 1 h. After washing with 1x PBS to remove unbound viruses, the levels of attached viruses were determined by measuring the cell-associated viral RNA by RT-qPCR. (**B**) Cell-ELISA was performed to measure the levels of viruses attached to the surface of Vero cells with the EV-A71 VP1 antibody. (**C**) RNA FISH to detect viral RNA in cells after exposure to EV-A71 for 10, 20, or 30 min. NH_4_Cl was used to neutralize lysosomes and thus block viral capsid uncoating and viral RNA release. (**D**) The numbers of visible red dots corresponding to EV-A71 RNA genomes per cell were shown. (**E**) Detection of viral RNA in Vero cells 30 min after infection with wild-type or mutated EV-A71 by RNA FISH. Nuclei are stained with DAPI in blue, the EV-A71 RNA genome is shown in red, EV-A71 capsid is shown in green. Representative images are shown. Scale bars: 10 µm. (**F**) The numbers of visible red dots corresponding to EV-A71 RNA genomes per cell were shown. (**G**) Vero cells were infected with wild type or mutated EV-A71 at 37°C for 1, 3, and 6 h before the levels of viral negative-strand RNA were determined by RT-qPCR with a tagged RT primer. Error bars represent SEM. ****, *P* < 0.0001. ns, not significant.

The lethal phenotype of the above VP1 N-terminal mutations in re-infection prompted us to investigate the effects of these mutations on the early steps of EV-A71 infection, from virus attachment to the cell surface until capsid uncoating and release of viral genomic RNA into the cytoplasm for translation and replication. To examine EV-A71 attachment, we first produced the wild-type and mutated viruses by transfecting viral RNA into Vero cells, then concentrated the viruses through ultracentrifugation. Viruses of the same amount of viral RNA were used to incubate with cells at 4°C for 1 h, allowing virus binding to the cell surface but no entry. After thorough washing with ice-cold PBS to remove unbound viruses, the bound EV-A71 was determined by measuring the levels of viral RNA. The results of RT-qPCR showed that the N71A mutant was 90% impaired in attaching to cells, mutants M63A, E65A, T66A, L70A, and N71A were 50% lower compared with the wild-type of EV-A71, mutants T32A and A46T were not affected (Fig. 5A). Next, we assessed EV-A71 attachment by performing Cell-ELISA to measure the levels of absorbed viruses; again, wild type-level attachment was observed for mutants T32A and A46T (Fig. 5B).

We next measured the release of viral RNA into the cytoplasm by the wild type and mutated EV-A71. We first performed an RNA fluorescence *in situ* hybridization (FISH) experiment to detect EV-A71 RNA in cells exposed to the wild-type virus for 10, 20, and 30 min. Viral VP1 was detected at 10 min and 20 min after infection at 37°C, indicating successful internalization of virus particles (Fig. 5C). ImageJ was used to analyze the number of EV-A71 genomic RNA puncta per cell (Fig. 5D). An average of 2.2 and 5.2 puncta per cell were observed at the two respective time points, suggesting that EV-A71 was not efficiently uncoated at this time point. Although at 30 min after infection, an average of 15.7 puncta per cell was observed. The amount of EV-A71 genomic RNA significantly increases by around three times at 30 min compared with that at 20 min. Moreover, EV-A71 RNA and capsid double-labeled vesicles, and EV-A71 RNA abutting capsid-positive structures underwent clustering at 30 min, suggesting uncoating more efficiently occurs at this time point (Fig. 5C). To verify that the observed viral RNA signals are a result of viral uncoating, we added NH_4_Cl to neutralize lysosomes and block viral capsid uncoating (33), almost no viral RNA was detected (Fig. 5C and D). We next exposed cells to the wild-type and mutated EV-A71 for 30 min at 37°C, then performed RNA FISH to detect the released viral RNA. As opposed to wild-type EV-A71 that completed viral capsid uncoating and viral RNA release, almost none of the mutants produced detectable viral RNA under these conditions (Fig. 5E). ImageJ was used to analyze the number of EV-A71 genomic RNA puncta per cell (Fig. 5F). This result is consistent with the above-mentioned results. In support of the defect in viral RNA release, at 6 h after infection, when the wild type of EV-A71 began to synthesize its negative-strand RNA, none of the viral mutants produced detectable amounts of viral negative-strand RNA (Fig. 5G). Together, these data suggest that these viral mutants are defective in delivering viral RNA into target cells for replication, corroborating their inability to reinfect and spread in cultured cells.

We summarized the effects of the VP1 N-terminal mutations on viral RNA replication, viral protein synthesis, virus particle production, virus attachment to target cells, and viral RNA release from the uncoated viral capsid in Table 1. Although all severe mutations block viral RNA release, mutations closer to the C-terminal end of this short region also exert a deleterious effect on virus production and attachment to the cell surface.

**TABLE 1:** Summary of the function of the N-terminal mutations^*a*^

VP1	Viral RNA replication	Viral protein synthesis	Virus particle production	Virus attachment		Virus RNA release	
	12 h	12 h	12 h	RT-qPCR	Cell-ELISA	RNA	Image
WT	1.0	1.0	1.0	1.0	1.0	1.0	++
T32A	0.75	0.78	1.17	0.85	0.97	0.03	–
A46T	1.02	0.93	0.47	1.6	0.96	0.01	–
M63A	0.91	1.07	0.71	0.49	0.58	0.01	–
E65A	0.58	0.73	0.69	0.51	0.16	0.00	–
T66A	0.79	1.17	0.46	0.52	0.18	0.03	–
L70A	0.67	0.99	0.20	0.38	0.12	0.07	–
N71A	0.41	0.78	0.11	0.15	0.14	0.03	–

^*a*^ ++: ~50% positive signal; –, no signal detected.

### Structural basis of uncoating deficiency

**Fig 6:**
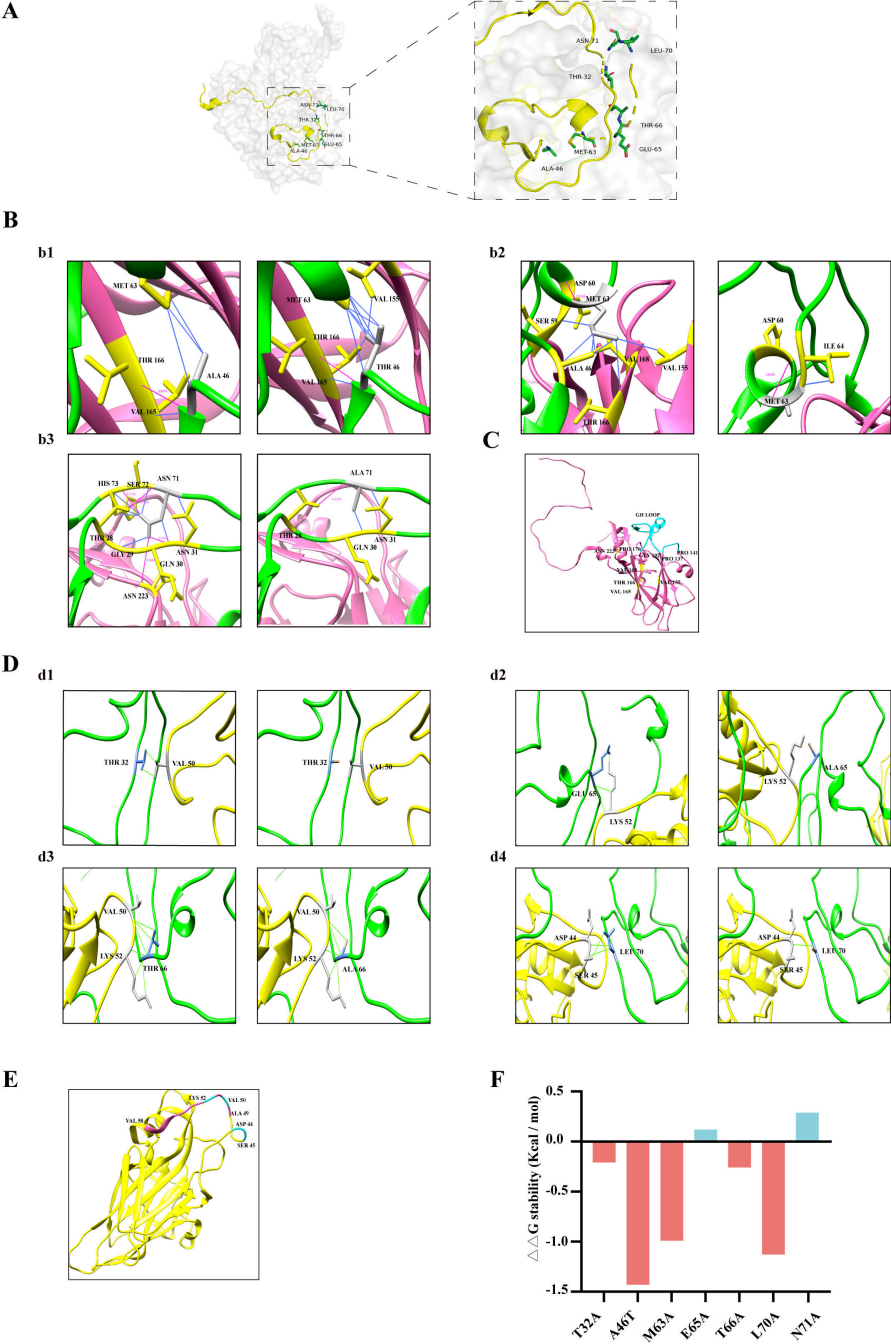
Structural modeling to predict the effect of VP1 mutations on the interaction networks and stability of EV-A71 capsid protomer. (**A**) The monomer crystal structure of EV-A71 (PDB: 3VBS) is shown. The N-terminal region is highlighted in yellow, with the seven highly conserved residues depicted in green. (**B**) Changes in hydrogen bonds and hydrophobic interactions at the VP1–VP3 interfaces are noted. The VP1 protein (green) and VP3 protein (hot pink) are presented as ribbons. Simulated mutations in VP1 residues 46, 63, and 71 (gray sticks) disrupted hydrogen bonds (magenta lines) and hydrophobic interactions (blue lines) with VP1 residues 28–31, 46, 59–60, 63–64, 72–73 (yellow sticks) and VP3 residues 155, 165, 166, 168, and 223 (yellow sticks). (**C**) The VP3 protein (hot pink) is displayed as ribbons. The VP3 GH loop (residues 170–192) is represented in cyan. The residues 155, 165, 166, and 223 (yellow) in VP3 were simulated. (**D**) In the VP1 (green)-VP2 (yellow) interface, simulated mutations in VP1 residues 32, 65, 66, and 70 (blue sticks) disrupt hydrophobic interactions (green lines) with VP2 residues 44, 45, 50, and 52 (gray sticks). (**E**) The VP2 protein (yellow) is displayed as ribbons. The VP2 (residues 49–58) is shown in hot pink. VP2 residues 44, 45, 50, and 52 (cyan) were simulated. (**F**) The predicted values of changes in folding free energy (ΔΔG) for substituted amino acids are provided.

To understand the key role of the highly conserved amino acids in the N-terminal region of VP1 in viral capsid uncoating and viral RNA release, we analyzed the hydrogen bond and hydrophobic networks involving these conserved VP1 residues using protein modeling on the UCSF Chimera with the available templates predicted that effects of these mutations on the structure of capsid protomer consisting of VP1, VP2, VP3, and VP4. We selected the RCSB Protein Data Bank (PDB: 3VBS) as the structural model because the amino acid sequence identity was above 98%. The N-terminal domain of the VP1 protein is positioned along the interior surface of the capsid (26). Spatial distribution of the seven highly conserved residues is highlighted in Fig. 6A. As the hydrogen-bond and hydrophobic interactions play a crucial role in the structure and function of proteins, we primarily investigated the hydrogen-bond and hydrophobic patterns associated with the mutated residues (Table 2). Effects of mutating these amino acids in the context of the capsid protomer were simulated using UCSF Chimera. The results separated these mutations into two clusters. Cluster I includes mutations A46T, M63A, and N71A that are positioned in close proximity to VP3 GH Loop. A46T increases the hydrogen bond with VP3–V165 while forming 10 hydrophobic interactions (VP1–M63, VP3–V165) (Fig. 6Bb1). This rearrangement is expected to rigidify the VP1–VP3 interface and may prevent the GH loop from conformational switching (residues 170–192), which is required for particle expansion. M63A disrupts seven hydrophobic bonds (VP1–A46/S59) and (VP3–V155/T166/V68), destabilizes the VP3 GH loop hinge region (Fig. 6Bb2). N71A eliminates three hydrogen bonds (VP1–E30/H73, VP3–N223) and five hydrophobic contacts (VP1–G29/S72/H73), thus weakens intra- and inter-protomer stability (Fig. 6Bb3). It is known that the VP3 GH loop undergoes a conformational switch with residues 170-192 converting from loop and helix to almost a β-hairpin upon capsid expansion and becoming less ordered (11). As shown in Fig. 6C, the residues V165, T166, and V168 are close to the residue of P170 located at the VP3 GH loop domain. It is likely that mutations A46T and M63A obstruct channel opening, fail to form the expanded particle, and hinder the release of the virus RNA.

**TABLE 2:** Comparison of mutation-mediated changes in hydrogen-bond networks in VP1

VP1 substitution	H-bound	Donor	Acceptor	Distance (Å)	Hydrophobic	Donor	Acceptor	Distance (Å)
Wild type	32THR-A:68CYS	O	SG	3.759	32THR-A:33GLN	CG2	O	3.549
	32THR-A:69VAL	O	N	2.873	32THR-A:66THR	CG2	CB	3.599
	32THR-A:69VAL	N	O	3.028	32THR-A:67ARG	OG2	O	3.267
					32THR-A:68CYS	O	CA	3.244
					32THR-A:69VAL	CB	CB	4.021
					32THR-B2:50VAL	CG2	CB	3.829
					32THR-B2:50VAL	OG1	CG1	3.384
	46ALA-C:166THR	O	N	2.937	46ALA-A:63MET	CB	SD	3.563
					46ALA-A:63MET	CA	SSD	3.790
					46ALA-A:63MET	CB	CD	3.871
					46ALA-C:165VAL	O	CA	3.467
	63MET-A:60ASP	N	O	2.916	63MET-A:46ALA	SD	CA	3.790
					63MET-A:46ALA	SD	CB	3.563
					63MET-A:46ALA	CE	CB	3.871
					63MET-A:59SER	CG	O	3.543
					63MET-C:155VAL	CE	CG2	3.510
					63MET-C:166THR	CE	O	3.307
					63MET-C:168VAL	CG	CG2	3.612
					65GLU-A:35SER	CA	OG	3.229
					65GLU-A:36SER	CG	CA	4.021
					65GLU-A:36SER	CD	CA	4.049
					65GLU-A:37HIS	CG	N	3.787
					65GLU-A:37HIS	CD	N	3.574
					65GLU-B2:52LYS	OE1	NZ	3.354
					65GLU-B2:52LYS	O	CB	3.194
					65GLU-B2:52LYS	O	N	2.896
	66THR-A:33GLN	OG1	O	2.598	66THR-A:34VAL	O	CA	3.379
	66THR-A:35SER	O	N	3.062	66THR-A:35SER	OG1	CB	3.529
	66THR-A:35SER	N	OG	2.788	66THR-A:61GLU	OG1	CG	3.583
	66THR-A:35SER	OG1	OG	3.264	66THR-A:61GLU	CG2	CA	4.056
					66THR-A:64ILE	CG2	CG1	3.674
					66THR-A:64ILE	CG2	CD1	3.926
					66THR-A:64ILE	N	O	3.205
					66THR-B2:50VAL	CG2	O	3.557
					66THR-B2:50VAL	CB	O	3.156
					66THR-B2:50VAL	CA	O	3.199
					66THR-B2:52LYS	CA	N	3.626
					66THR-B2:52LYS	C	CB	3.613
					66THR-B2:52LYS	O	CE	3.489
					LEU70-A:31ASN	CD2	ND2	3.362
					LEU70-A:31ASN	CA	ND2	3.589
					LEU70-B2:44ASP	N	O	2.799
					LEU70-B2:45SER	CD1	CB	4.054
					LEU70-B2:45SER	CB	CA	3.958
	71ASN-A:28THR	O	OG1	3.313	71ASN-A:29GLY	ND2	CA	3.663
	71ASN-A:30GLN	ND2	O	3.167	71ASN-A:30GLN	CB	O	3.268
	71ASN-A:73HIS	OD1	N	2.717	71ASN-A:31ASN	N	CG	3.539
	71ASN-C:223ASN	ND2	O	3.292	71ASN-A:72SER	OD1	N	2.939
					71ASN-A:73HIS	OD1	CA	3.453
					71ASN-A:73HIS	OD1	CG	3.119
					71ASN-A:73HIS	OD1	CB	3.078
T32A	32THR-A:68CYS	O	SG	3.759	32THR-A:68CYS	O	CA	3.244
	32THR-A:69VAL	O	N	2.873	32THR-A:69VAL	CB	CB	3.988
	32THR-A:69VAL	N	O	3.028				
**A46T**	46THR-C:166THR	O	N	2.937	46THR-A:63MET	CA	SD	3.790
					46THR-A:63MET	CB	SD	3.573
					46THR-A:63MET	CG2	SD	3.851
					46THR-A:63MET	CG2	CE	3.521
					46THR-A:63MET	CB	CEE	3.879
					46THR-A:63MET	OG1	SD	2.643
					46THR-A:63MET	OG1	CE	3.133
					46THR-C:155VAL	CG2	CG1	3.727
					46THR-C:165VAL	O	CA	3.467
					46THR-C:165VAL	CG2	CG2	3.196
M63A	63ALA-A:60ASP	N	O	2.916	63ALA-A:64ILE	O	CG2	2.969
E65A					65GLU-A:35SER	CA	OG	3.229
T66A	66THR-A:35SER	O	N	3.062	66THR-A:32THR	CB	CG2	3.603
	66THR-A:35SER	N	OG	2.788	66THR-A:34VAL	O	CA	3.379
					66THR-A:64ILE	N	O	3.205
					66THR-B2:50VAL	CB	O	3.156
					66THR-B2:50VAL	CA	O	3.199
					66THR-B2:52LYS	CA	N	3.626
					66THR-B2:52LYS	C	CB	3.613
					66THR-B2:52LYS	O	CE	3.489
					ALA70-A:31ASN	CA	ND2	3.589
L70A					ALA70-B2:44ASP	N	O	2.799
N71A	71ASN-A:28THR	O	OG1		71ASN-A:30GLN	CB	O	3.276
					71ASN-A:31ASN	N	CG	3.539

Cluster II includes mutations T32A, E65A, T66A, and L70A that may affect VP2-mediated 2-fold channel dynamics. T32A disrupts five hydrophobic bonds with VP2–V50 and VP1-Q33/T66/R67, thus destabilizing the VP1–VP2 interface (Fig. 6Dd1). E65A/T66A weakens several critical hydrophobic linkages between VP1 (S35/36, I64) and VP2 (K52/V50), thus undermining the function of the VP2 bridge (residues 48–52) in stabilizing the 2-fold channel (Fig. 6Dd2). L70A abolishes three interactions (VP1–N31, VP2–S45), compromising VP1–VP2 connectivity (Fig. 6Dd3). Given the known function of VP2 bridge (residues 48–52) in viral capsid expansion (11, 16) (Fig. 6E), these data suggest that VP1 amino acids T32, E65, T66, and L70 may be critical for maintaining the VP2 bridge at the site of the 2-fold channel, which needs to open for the exit of viral RNA.

To further examine the possible effects of these VP1 N-terminal mutations on structural stability, we used DynaMut2 to predict the ΔΔG values with each of these mutations. The results showed that mutations T32A, A46T, M63A, T66A, and L70A lower ΔΔG < −0.21 kcal/mol, thus exerting destabilizing effect and reducing rigidity of the VP1 N-terminal region (Fig. 6F). Mutations E65A and N71A significantly increase ΔΔG < 0.5kcal/mol, thus promoting flexibility (Fig. 6F). These energy shifts likely hinder the coordinated movement of the VP1 N-terminal region that is critical for capsid expansion and RNA release.

## DISCUSSION

In this study, we systematically investigated the functions of the 21 highly conserved amino acids in the VP1 protein. In support of their highly conserved nature, we found that only the amino acid at position 168 is dispensable for EV-A71 replication, a single mutation of 12 highly conserved amino acids is lethal to the virus, and mutations of the rest of the eight conserved amino acids significantly cripple the virus. Importantly, the seven highly conserved amino acids in the 71-amino-acid N-terminal region are all indispensable for the infectivity of EV-A71, and no viral reversion was observed after 5 passages of these seven viral mutants. These results are in agreement with previous findings of mutations T75A, T78A, G88A, and A107T that inhibit EV-A71 infection by impairing the functions of the viral capsid (31, 32).

VP1 makes a major contribution to cell attachment and receptor binding, and some of these highly conserved amino acids are located in VP1 loops that interact with cell surface factors. For example, VP1 amino acids K242 and K244 mediate viral capsid attachment to the cell surface through binding to PSGL-1 and/or heparan sulfate (34, 35). The VP1-G/Q/E145 amino acid acts as a molecular switch to control PSGL-1 binding by the modulation of the exposure of VP1–K244 (34). Residues G99, G105, A139, T141, and S168 are proximal to the site of binding to heparan sulfate (36). Mutations of these amino acids may have impaired the attachment of EV-A71 capsid to the cell surface, thus diminishing viral infection. Although our data suggest that the substitution of serine at position 168 with alanine does not affect EV-A71 replication, the importance of highly conserved S168 awaits more extensive mutagenesis and functional characterization. The scavenger receptor B2 (SCARB2) plays a role in the binding, internalization, and uncoating of EV-A71 and is expressed in all tissues (10, 37, 38). Chen et al. have shown that R166A and R236A mutations reduced SCARB2 binding and infection of RD cells but did not interfere with virus assembly (27). We found that amino acids G105, P158, G159, A160, and S168 are located in the binding site of receptor SCARB2 (Fig. 1E). The lethal phenotype of mutations G105A and A160T and the defective phenotype of mutations P158A and G159A might have been a result of the detrimental effect of these mutations on viral capsid binding to SCARB2. Interestingly, amino acid G105 is also located at the heparan sulfate binding site, suggesting its role in virus attachment. Amino acids Q118, A133, P158, G159, and A160 are located close to the VP1 pocket structure (Fig. 1F). Their mutations may hinder pocket collapse and dislodgement of pocket factors, which are an essential step during capsid uncoating. Notably, some residues may be involved in more than one function, such as amino acids P158, G159, and A160 that are predicted to participate in SCARB2 binding and pocket rearrangement. Thus, mutating these amino acids may impair multiple functions of VP1 and the viral capsid.

Mutations of the seven highly conserved amino acids, T32A, A46T, M63A, E65A, T66A, L70A, and N71A, in the N-terminal region of VP1 abrogate the production of replicative EV-A71 viruses (Fig. 3A through F). This drastic phenotype is in line with the key roles of this N-terminal region in capsid assembly and capsid uncoating, as reported (8, 18, 39, 40). The results of structural modeling of EV-A71 capsid protomer revealed that these seven amino acids are involved in the intricate interaction networks with VP2 in the adjacent protomer and VP3 in the same protomer (Fig. 6A through D). Not surprisingly, mutating these amino acids significantly alters these interactions. For example, mutations A46T, M63A, and N71A disrupt VP3 GH loop dynamics through altering hydrogen bonding and hydrophobic interactions. Given the significant structural change of the VP3 GH loop region (residues 170–192) from loop and helix to almost β-hairpin during capsid expansion (11), these three mutations may hinder the function of the VP3 GH loop in the efficient uncoating of EV-A71. The other four mutations, T32A, E65A, T66A, and L70A, disrupt VP1 interaction with the VP2 bridge region from the neighbor protomer (Fig. 6D). Since this VP2 bridge regulates channel opening for RNA release when the native particles expand (11, 16), these four mutations may prevent the release of viral RNA. These defects in viral capsid uncoating and viral RNA release were all observed with these seven N-terminal mutations in our experiments (Fig. 5E). Notably, these mutations had no significant effects on viral RNA transcription and viral protein synthesis (Fig. 4A and B), whereas mutations L70A and N71A diminished production of virus particles (Fig. 4C and D), suggesting a role of these two amino acids in viral assembly.

Although the N-terminal region of VP1 is not directly implicated in virus binding to cell surface proteins because this region is not located in the canyon structure in the viral capsid, our data do show that mutants M63A, E65A, T66A, L70A, and N71A had lower attachment to the cell surface (Fig. 5A and B). We speculated that these mutations might have altered the structure of the distal receptor-binding region in the canyon through disrupting the network of bonding within and between viral capsid protomers, as suggested by our structure modeling data in Fig. 6. This possibility awaits structural determination of the mutated viral capsid using techniques such as Cryo-ET.

In summary, we have systematically characterized the highly conserved amino acids in viral protein VP1 and demonstrated their essential role in the production of infectious EV-A71 particles. Specifically, our data further support the crucial roles of the N-terminal region of VP1 in viral capsid uncoating and viral RNA release. Mutating these highly conserved amino acids in VP1 can be a strategy for developing attenuated EV-A71 vaccine candidates, given the impaired viral infectivity and the lack of viral reversion. Positions of these highly conserved amino acids may also guide the design and test of small molecule compounds that target VP1 and viral protomer and block viral capsid function and viral replication. One successful example of this type of inhibitor is lenacapavir, which binds to HIV-1 capsid and blocks the production of infectious viral particles (41–43). This represents a promising avenue for developing EV-A71 inhibitors.

## MATERIALS AND METHODS

### Cell culture

African green monkey kidney cells (Vero) were purchased from ATCC (CCL-81). The cells were cultured in Dulbecco’s modified Eagle’s medium (DMEM) (Gibco, Carlsbad, CA) supplemented with 10% fetal bovine serum (FBS) with 5% CO_2_ at 37°C.

### Mutagenesis

A total of 3,777 EV-A71 strains were analyzed from the GenBank database, revealing 21 amino acid residues that remained invariant among all strains. All mutations in the VP1 gene were constructed via PCR using the plasmid EV-A71-HP (genotype C4) as the template (GenBank accession no. KY074643) (31). The sequences of primers used for site-directed mutagenesis are as follows:

T32A, forward primer 5′-CAGCACCCACAGGCCAGAACGCACAGGTGAGCAGTCATCGA-3′ and reverse 5′-TCGATGACTGCTCACCTGTGCGTTCTGGCCTGTGGGTGCTG-3′; A46T, forward primer 5′-TGGATACAGGCAAGGTTCCAACACTCCAAGCTGCTGAAATT-3′; reverse 5′-AATTTCAGCAGCTTGGAGTGTTGGAACCTTGCCTGTATCCA-3′; M63A, forward primer 5′-CAAATGCTAGTGACGAGAGCGCGATTGAGACACGCTGTGTTC-3′; reverse 5′-GAACACAGCGTGTCTCAATCGCGCTCTCGTCACTAGCATTTG-3′; E65A, forward primer 5′-TAGTGACGAGAGCATGATTGCGACACGCTGTGTTCTTAACT-3′; and reverse, 5′-AGTTAAGAACACAGCGTGTCGCAATCATGCTCTCGTCACTA-3′; T66A, forward primer 5′-GTGACGAGAGCATGATTGAGGCACGCTGTGTTCTTAACTCG-3′; reverse 5′-CGAGTTAAGAACACAGCGTGCCTCAATCATGCTCTCGTCAC-3′; L70A, forward primer 5′-TGATTGAGACACGCTGTGTTGCTAACTCGCACAGTACAGCTG-3′; reverse 5′-CAGCTGTACTGTGCGAGTTAGCAACACAGCGTGTCTCAATCA-3′; N71A, forward primer 5′-TTGAGACACGCTGTGTTCTTGCCTCGCACAGTACAGCTGAGA-3′; reverse 5′-TCTCAGCTGTACTGTGCGAGGCAAGAACACAGCGTGTCTCAA-3′. The mutated EV-A71 viral genome was confirmed by Sanger sequencing.

### *In vitro* transcription and RNA transfection

The cDNA plasmids were linearized with *HinDIII* digestion and purified by a DNA clean-up kit (Magen, D2111-03). *In vitro* RNA transcription was performed with a T7 transcription kit (Hongene Hiotech, ON-040) following the manufacturer’s instructions. In total, 2 µg RNA was transfected into 2 × 10^5^ Vero cells using 5 µL Lipofectamine RNAiMAX reagent (Thermo Fisher Scientific, 13778150). Viruses were harvested after transfection, and aliquots were stored at −80°C.

### Western blot analysis

Cell or virus samples were denatured in 1× SDS loading buffer at 100°C for 15 min. Proteins were separated using 10% SDS-PAGE and then transferred onto the polyvinylidene difluoride (PVDF) membrane. The membrane was blocked with 5% non-fat milk for 1 h at room temperature and washed with 1 × PBST (0.1% Tween-20). Membranes were then incubated with the primary antibodies including anti-EV71 VP0/VP2 antibody (diluted 1:1,000 ～1:5,000), anti-EV71 VP1 antibody (diluted 1:1,000 ～1:5,000), or anti-β-actin antibody (diluted 1:5,000) at 4˚C overnight. After washing three times with 1 × PBST, HRP-conjugated secondary antibody (1:5,000 dilution) was added for 2 h at room temperature. Protein signals were detected using Western Lighting chemiluminescence reagent (Millipore).

### RNA extraction and quantitative RT-PCR (qRT-PCR)

Total RNA was extracted from cells or the culture supernatant using Trizol or Trizol LS reagents (Invitrogen) according to the manufacturer’s instructions. cDNA was synthesized by reverse transcription in a reaction mixture containing 2 µM of specific primers at 42°C for 1 h using M-MLV RT kit, according to the manufacturer’s instructions (Promega, M170B). The cDNA levels were determined by real-time PCR in a 20 µL reaction mixture containing 0.5 µL of cDNA product, 0.4 µL of forward and reverse primers, and 10 µL of Power SYBR Green PCR Master Mix (Vazyme, Q711-02), according to the manufacturer’s instructions. The data were collected by QuanStudio Design & Analysis software (version 1.5.1). Relative mRNA levels were determined using the comparative 2^−∆∆Ct^ method by normalizing them to GAPDH or generating a relative standard curve using a serial dilution of the plasmid DNA. The primers used in the real-time qRT-PCR are as follows: EV-A71-VP1 real-time PCR forward 5′-CGCCACTAACCCCTCAGTTT-3′ and reverse 5′-AGTTCTGGTTACGCATCGGG-3′, and GAPDH real-time PCR forward 5′-GTCCACTGGCGTCTTCACCA-3′ and reverse 5′-GTGGCAGTGATGGCATGGAC-3′.

### Negative strand-specific quantitative RT-PCR

A tagged forward primer Tag-3D-F (5′-CTCGGCCAACTCTGATGAAGCCCTATAGTGCCCTGGGGAT-3′) was designed containing a 20-nucleotide Tag sequence unrelated to EV-A71 at the 5’-end with the rest of the primer sequence (20 bases) specific to the EV-A71 negative strand. The cDNA was produced using the M-MLV reverse transcriptase with Tag-3D-F primer (44). Reverse transcription was performed at 50℃ for 40 min, followed by 10 min at 95℃. For the quantification of the negative strand RNAs, real-time quantitative PCR was performed with the primer sets of Tag (5′-CTCGGCCAACTCTGATGAAG-3′) and 3D-R (5′-CGCAGCTCGTCCTTGACATA-3′) using ChamQ SYBR qPCR Master Mix (Vazyme, Q331), following the manufacturer’s instructions.

### TCID_50_ assay

Vero cells were seeded in 96-well plates at 1 × 10^4^ cells/well for 24 h before they were infected with serial dilutions of EV-A71. After 7 days of incubation at 37°C with 5% CO_2_, wells exhibiting with CPE were scored. Values of 50% tissue-culture effective dose (TCID_50_) were calculated according to the Reed-Muench method.

### RNA fluorescence *in situ* hybridization assay

Cells were infected with EV-A71 viruses and mutants at 4°C for 1 h. Infected cells were washed three times with PBS, with or without NH_4_Cl, and incubated at 37°C for internalization, followed by fixation with 4% PFA at room temperature for 15 min at the indicated times. After washing three times with 1 × PBST, the cells were incubated with mouse anti-EV-A71-VP1 monoclonal antibody (Invitrogen, MA5-33258) at 4°C overnight, and then incubated with FITC-conjugated goat anti-mouse secondary antibodies (1:1,000) for 1 h. Cells were further fixed in 4% PFA at room temperature for 15 min. The cells were then treated with RNA fish wash buffer A reagent (LGC, SMF-WA1-60) for 2–5 min and incubated with RNA probes (LGC) at 37°C for 16 h. The cells were treated again with RNA fish wash buffer A reagent (LGC, SMF-WA1-60) at room temperature for 30 min. The nuclei were counterstained with 4′, 6′-diamidino-2-phenylindole (DAPI) for 5 min. Then the cells were treated with RNA fish wash buffer B (LGC, SMF-WA1-20) for 2–5 min. The cells were observed under a fluorescence microscope FV-3000 (Olympus, Tokyo, Japan).

### Cell-based ELISA

Vero cells were seeded in 96-well plates at 1 × 10^4^ cells per well for 24 h before they were incubated with EV-A71 at 4°C for 1 h. After three times washing with 1xPBST to remove unbound viruses, cells were fixed with 4% PFA at room temperature for 20 min. Fixed cells were blocked in 5% non-fat milk for 1 h at room temperature, then incubated with primary antibodies of anti-EV71-VP1 (Abnova, MAB1255-M08, 1:100) for 1 h at 37°C. After washing three times with 1 × PBST, the horseradish peroxidase (HRP)-conjugated secondary antibody (ZSGB-BIO, ZB-2305, 1:1,000) was added at room temperature for 1 h. TMB peroxidase substrate (Solarbio, PR1210) was added for 1 h before the stop solution was added. The absorbance was recorded at OD 450 nm using a microplate reader.

### Attachment assay

Viruses of the same amount of genomic RNA were incubated with Vero cells at 4°C for 1 h for the viruses to attach to the cell surface. The unbound viruses were removed by washing in 1 × PBST. Total RNA was extracted from the treated cells, and the amount of EV-A71 RNA was determined by qRT-PCR as described above.

### Interaction bonds network analysis

The structure of the N-terminal of the VP1 protein was extracted from the mature EV-A71 particle (PDB: 3VBS) by Pymol software. To predict the effect of the mutations on the structure of the capsid protomer, a protein model was established by UCSF Chimera and was used to visualize and analyze the three-dimensional protein structure and alteration of hydrogen bonds and hydrophobic interactions. The stability of the mutated VP1 structure was predicted using the website of the Dydamut2 algorithm (45).

### Statistics

All experiments were performed with at least three biological duplicates. Data are presented with error bars indicating standard deviations. Unpaired, two-tailed Student’s *t*-test analyzed comparisons between individual and corresponding control groups. A one-way analysis of variance with Dunnett (selected pairs) post hoc test was conducted to compare the results obtained from the different experimental groups. GraphPad Prism (version 9.5.0; GraphPad Software, USA) was used for statistical analyses, with a *P* value of <0.05 indicating significance.

## Data Availability

All data required for evaluating the conclusions of this study are available within the paper or from the authors upon request.
